# To what extent does access to improved sanitation explain the observed differences in infant mortality in Africa?

**DOI:** 10.4102/phcfm.v9i1.1370

**Published:** 2017-05-29

**Authors:** Aye M. Alemu

**Affiliations:** 1 SolBridge International School of Business, Woosong University, Republic of Korea

## Abstract

**Background:**

To my knowledge, there was no systematic study so far that analysed the extent of the impact of improved sanitation on infant mortality in the African context with long years of full-fledged longitudinal data.

**Aim:**

The aim of this study was to empirically examine the extent to which improved sanitation explains the observed differences in infant mortality under 5 years of age across African countries.

**Setting:**

The study covered a panel of 33 countries from north, south, east, west and central Africa for the years 1994–2013.

**Methods:**

The study first conducted Durbin–Wu–Hausman specification test and then used fixed effect model. In addition, Praison–Winsten regression with corrected heteroscedasticity was employed to verify the consistency of the results that were revealed in using fixed effect estimation method.

**Results:**

The study revealed that a 1% increase in access to improved sanitation would reduce infant mortality by a rate of about two infant deaths per 1000 live births. Also, the study confirmed that a significant decline in infant mortality rate was highly linked to improvements in education, health and sustainable economic growth.

**Conclusion:**

The findings have wide implications especially for African countries for which decreasing infant mortality is one of the most crucial priorities in the continent to reverse the current deep-rooted challenges related to human capital formation.

## Introduction

‘An ounce of prevention is better than a pound of cure.’ (Benjamin Franklin)

Putting this quote into context means that improved access to water and sanitation, which are part of basic necessities of human beings, may save millions of infants from dying before the age of 5 years and beyond. Infant mortality is a sensitive indicator of social progress and disparities. About 8.8 million children die every year around the world before their fifth birthday.^1^ By the same token, about 29 000 children under the age of 5 every day or 21 children each minute die mainly from preventable causes. The situation is particularly dire in Africa where infant and child mortality rates are 15 times higher than that in high-income regions.^2^

Child mortality has been declining worldwide as a result of socioeconomic development and implementation of child-survival interventions. The aim of UN Millennium Development Goal 4 (MDG 4) is to reduce mortality of children younger than 5 years by two-thirds between 1990 and 2015, but many countries, especially in south Asia and sub-Saharan Africa, have not met the target. Southern Asia continues to have both a high rate of under-five mortality, at 50 deaths per 1000 live births in 2015, and a large number of total deaths, at 1.8 million.^3^ In sub-Saharan Africa, the infant mortality rate fell from 90 deaths per 1000 live births in 1990 to 54 deaths per 1000 live births in 2014.^4^ This performance is promising, but still a long along way to go. According to WHO,^5^ under-five deaths are increasingly concentrated in sub-Saharan Africa and southern Asia, while the proportion in the rest of the world dropped from 32% in 1990 to 18% in 2013. Children in sub-Saharan Africa are more than 15 times more likely to die before the age of 5 than children in developed regions. According to UNICEF,^2^ Africa has made limited progress in providing its people with access to basic sanitation. Coverage only increased from 35% in 1990 to 40% in 2010, equal to 189 million people gaining access. However, with a population growth of 400 million people since 1990, the population without an improved sanitation facility increased by almost 200 million people to 612 million people in 2010.

According to a report compiled by United Nations University,^6^ in sub-Saharan Africa, on a typical day, half the hospital beds are occupied by people afflicted with faecal-borne disease, and treating preventable infectious diarrhoea consumes 12% of the total health budget. The same study further reveals improved sanitation in developing countries typically yields about $9 worth for every $1 spent.

Similar studies by Pruss-Ustun et al.^7^ suggest that almost one-tenth of the global disease burden could be prevented by improving water supply, sanitation, hygiene and management of water resources. Furthermore, Pruss et al.^8^ estimate that about 4.0% of all deaths and 5.7% of total disability-adjusted life years can be attributed to water, sanitation and hygiene. Worldwide, 1.4 million children die each year from preventable diarrhoeal diseases, and some 88% of diarrhoea cases are related to unsafe water, inadequate sanitation or insufficient hygiene.^9^

The most important causes of death in children younger than 5 years were infectious diseases, especially pneumonia, diarrhoea, preterm birth complications and malaria. Two-fifths of deaths occurred in the neonatal period, during which the greatest single causes of death were preterm birth complications and birth asphyxia, but collectively, infectious diseases were also important. A child’s risk of dying is highest in the neonatal period, the first 28 days of life. Safe childbirth and effective neonatal care are essential to prevent these deaths. Forty-four per cent of child deaths under the age of 5 take place during the neonatal period.^5^ The numbers of deaths varied widely across WHO regions, with most deaths recorded in Africa and southeast Asia.^10^

## Improved sanitation for reducing infant mortality

More than 2.6 billion people around the world – one in three – lack basic sanitation facilities.^7^ This results in four billion cases of diarrhoea each year and 400 million children chronically infected by intestinal parasites.^11^ Persistent diarrhoea and parasite infections result in chronic malnutrition, a compromised immune system, and impaired brain development. Whipworm infection accounts for half of all school absenteeism in the developing world. In fact, one study showed that 40% of worm infections resulted from unsanitary conditions at school.^2^ Accordingly, a study by UNICEF concluded that sanitation interventions were among the most cost-effective of all interventions in lowering the rate of death among children younger than 5 in the developing world, at a cost of about $11 per year of life saved.^12^ By the same token, more than half of under-five child deaths are because of diseases that are preventable and treatable through simple, affordable interventions.

**FIGURE 1:**
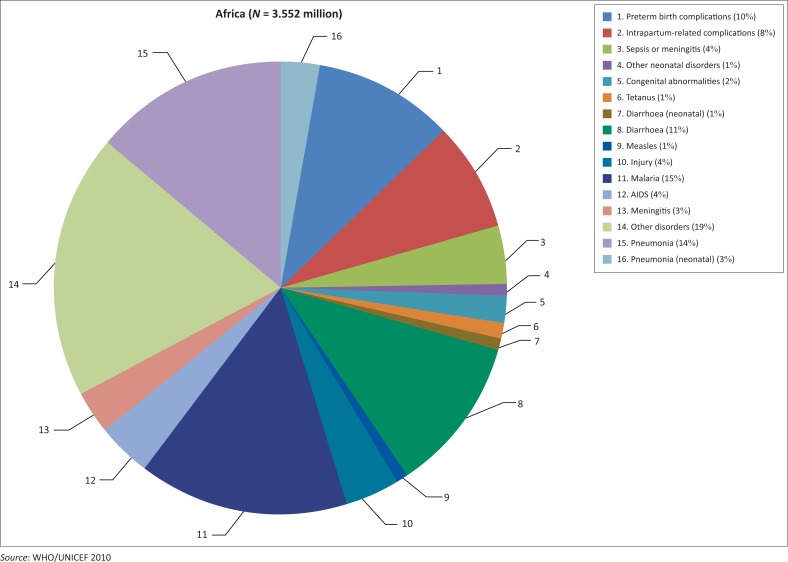
Causes of child deaths in Africa, 2010.

According to Black et al.,^10^ diarrhoea is now the biggest killer of children in Africa. Every day, 2000 African children die from diarrhoea – deaths that are entirely preventable. Nine out of ten cases of diarrhoea can be prevented by safe water and sanitation – proven cost-effective interventions. Despite this, today only four in ten Africans have access to a basic toilet. This failure will undermine efforts to accelerate progress on the MDG for child mortality. In Africa, access to safe water and effective sanitation is the lowest in all developing regions and coverage averages 58% for safe water and 31% for sanitation.^13^ Most of the causes of child deaths in Africa can be easily preventable by safe water and improved sanitation. Nevertheless, as clearly shown in Figure 1, about 29% of under-five deaths in Africa occur in the first 28 days of life, known as the neonatal period. As depicted, 1% of under-five deaths are caused by diarrhoea in the neonatal period, in addition to the 11% outside it. For pneumonia, these figures are 3% and 14%, respectively.

According to WHO/UNICEF,^14^ poor sanitation and water cause 28% of child deaths, and safe sanitation and water are proven and cost-effective interventions. On the other hand, safe sanitation and water could prevent nine out of ten cases of diarrhoea, and simply using a safe toilet can reduce the incidence of diarrhoea by nearly 40%. Safe sanitation also significantly reduces other leading causes of child deaths, such as under nutrition and pneumonia. Thus, addressing access to sanitation is key to reducing child mortality by two-thirds.

WHO/UNICEF^15^ indicates Africa has made limited progress in providing its people with access to basic sanitation. Coverage only increased from 35% in 1990 to 40% in 2010, equal to 189 million people gaining access. With a population growth of almost 400 million people since 1990, the population without an improved sanitation facility increased by almost 200 million people to 612 million in 2010. With a doubling of the urban population over the period 1990–2010, more than one in four people in urban areas rely on shared or public sanitation facilities. Little over one in five people in Africa still practise open defecation, down from one in three in 1990.

**FIGURE 2:**
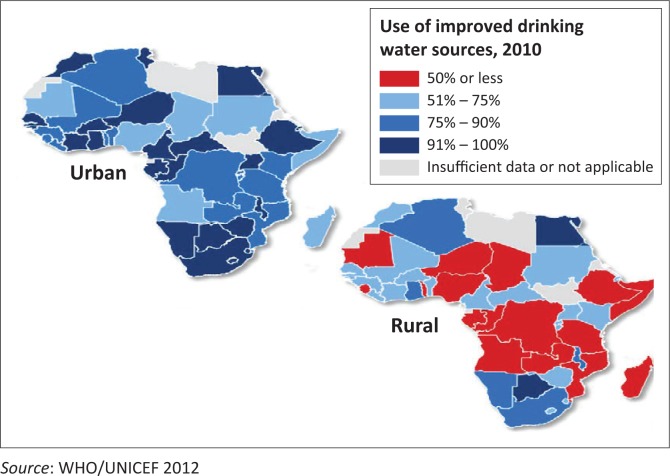
Use of improved drinking water in urban and rural African countries in 2010.

In Africa as a whole it is only South Africa, Botswana, Namibia and Egypt that have reached to a level of 91%–100% of coverage at national level in using improved drinking water sources. But, the most striking point is that the rural population that comprises the highest majority in almost all countries in Africa is by far lagging behind the urban areas in terms of having access to safe drinking waters (Figure 2).

**FIGURE 3:**
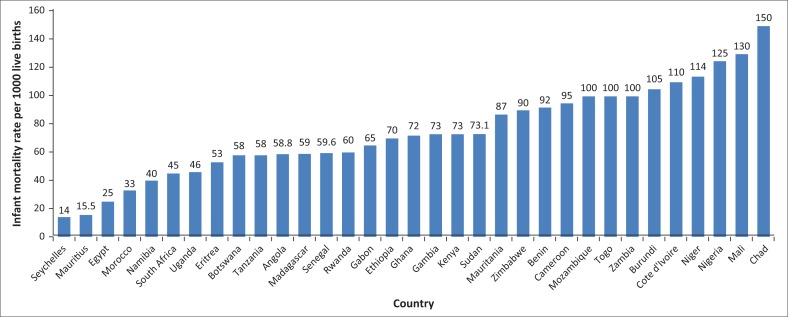
Infant mortality rate per 1000 lives of births in Africa in 2010.

**FIGURE 4:**
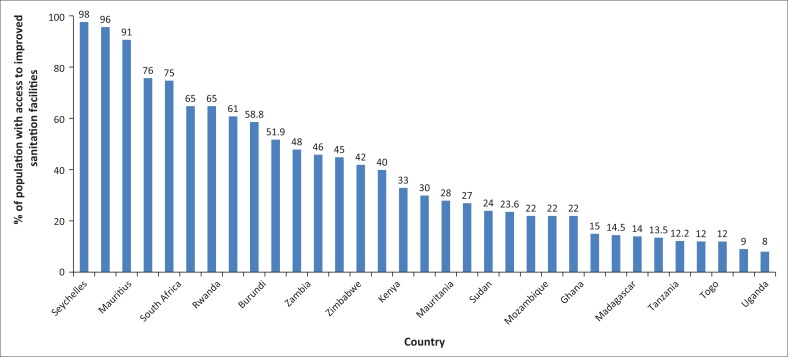
Improved sanitation facilities (percentage of population with access) in Africa in 2010.

Moreover, child mortality rate under 5 years per 1000 lives of birth and the level of improved sanitation facilities (percentage of population with access) in 2010 for about 33 African countries are shown in Figures 3 and 4, respectively. For instance, from Figure 3, it can be noted that Seychelles, Mauritius, Egypt, Morocco, Namibia and South Africa are the top six countries according to their order that substantially reduced infant mortality under 5 years of age. In Seychelles, for example, the infant mortality rate per 1000 live births is only about 14, which is absolutely exemplary in the continent. In contrast, countries with the highest infant mortality rate under 5 years of age according to their order include Chad, Mali, Nigeria, Niger, Cot devoir, Cote d’Ivoire and Burundi. Chad, for instance, experiences about 150 infant mortality rate under 5 years of age per 1000 lives of births.

Similarly, Figure 4 indicates the extent of improved sanitation in African countries and hence it has been shown that again Seychelles, Egypt, Mauritius, Morocco, South Africa and Botswana are the most successful African countries that were able to achieve a significant improvement in sanitation. On the contrary, countries such as Uganda, Niger, Togo, Chad, Tanzania, Eritrea, Madagascar and Benin have the most worrisome level of sanitation accessibility in Africa. For instance, while improved sanitation facilities (percentage of population with access) for Seychelles has been about 98%, this figure for Uganda was only 8% in 2010 (Figure 4).

## Objectives of the study

The main objective of this study is to systematically and empirically identify and analyse the extent to which improved sanitation influences infant mortality under the age of 5 in Africa. To my knowledge, there is no systematic study so far that has analysed the extent of the impact of improved sanitation on infant mortality under the African context with full-fledged longitudinal data. This study, therefore, will add knowledge to the existing literature in the field.

## Main research question

To what extent does access to improved sanitation explain the observed differences in infant and under-five mortality across African countries?

## Main propositions

This study hypothesises that improving sanitation would have a strong and significant effect in reducing infant mortality in Africa.

## The data and stylised facts

This study has made an intensive empirical analysis of a panel of 33 countries from north, south, east, west and central Africa for the years 1994–2013. The year 1994 was chosen as the starting point because of the availability of full, annual data covering the variables of interest for most African countries. The list of countries included in this study is shown in Box 1. These countries are selected mainly because of the availability of data throughout the years.

BOX 1 Countries included in the study.

Angola, Benin, Botswana, Burundi, Cameroon, Chad, Cote d’Ivoire, Egypt, Eritrea, Ethiopia, Gabon, Gambia, Ghana, Kenya, Madagascar, Mali, Mauritania, Mauritius, Morocco, Mozambique, Namibia, Niger, Nigeria, Rwanda, Senegal, Seychelles, South Africa, Sudan, Tanzania, Togo, Uganda, Zambia and Zimbabwe.

### Ethical consideration

Ethical clearance and approval were obtained from SolBridge Institutional Review Board (SIRB) on 21 March 2017. The review board confirmed that no human subjects were involved in this research, and only public panel secondary data were employed.

## Research methodology and estimation techniques

### Theoretical model for determinants of infant mortality at macro level

**FIGURE 5:**
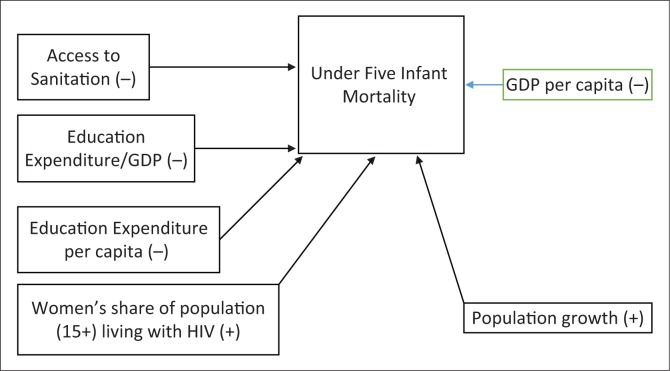
Theoretical model for determinants of infant mortality at macro level.

The theoretical model for determinants of infant mortality at macro level and their expected relationships are summarised in Figure 5. Access to sanitation is measured by the percentage of the population using improved sanitation facilities. Improved sanitation includes sewer connections, septic system connections, and pour-flush latrines, ventilated improved pit latrines and pit latrines with a slab or covered pit, etc. Thus, it has been hypothesised that improved sanitation, investment in education and health and sustainable economic growth captured by gross domestic product (GDP) per capita are expected to significantly contribute to a decrease in infant mortality under 5 years of age (hence all these four variables have negative signs to show their impacts are to decrease infant mortality). In contrast, women’s share of population (15+) living with HIV and population growth are expected to positively and significantly contribute to the increase in infant mortality under 5 years of age.

### The correlation between infant mortality and the independent variables

Although correlation does not mean causation, it is worth to examine the correlations that exist between ‘infant mortality’ and the independent variables before discussing the results of the regression analysis. In other words, the correlation coefficients of each variable determine the nature and strength of the relation between each factor and infant mortality. Accordingly, Table 1 indicates that improved sanitation access is negatively and highly correlated with infant mortality rate. In other words, the more the access to sanitation among societies, the less the infant mortality rate. Likewise, it is evident that the more the government spends on education and health, the lower the infant mortality rate. This is also true for the relationship between economic growth in a country and a reduction in infant mortality.

**TABLE 1:** Partial correlation of infant mortality under 5 years of age (per 1000 live births) with the independent variables.

Variable	Correlation coefficient	Significance level
Improved sanitation (percentage of population with access)	−0.2984	0.000*
Education expenditure or GDP	−0.3056	0.000*
Women’s share of population (15+) living with HIV	0.1750	0.000*
Health expenditure or GDP	−0.3226	0.000*
GDP/C (PPP)	−0.2875	0.000*
Population growth (annual)	0.5041	0.000*

GDP, gross domestic product; GDP/C, gross domestic product per capita; PPP, purchasing power parity.

* refer the level of statistical significance at 1 percent level.

In contrast, the correlation analysis from Table 1 shows that women’s share of population (15+) living with HIV and population growth in the respective countries are significantly and positively correlated with infant mortality rate. This implies that infant mortality in Africa is also highly related with HIV related diseases that may be transmitted from mother to-child despite a significant progress in reducing the incidence through the *prevention of mother-to-child transmission* (PMTCT) programmes. The fact that population growth has a direct and significant correlation may indicate that if population growth increases much faster than economic growth, it would lead to a significant reduction in investment in education and health that in turn would exacerbate infant mortality.

In fact, Figures 6–13 clearly demonstrate the strong and negative relationship between ‘improved sanitation’ and ‘infant mortality rate’ for selected African countries including Cote d’Ivoire, Egypt, Ethiopia, Ghana, Kenya, Mauritius, Morocco and Tanzania.

**FIGURE 6:**
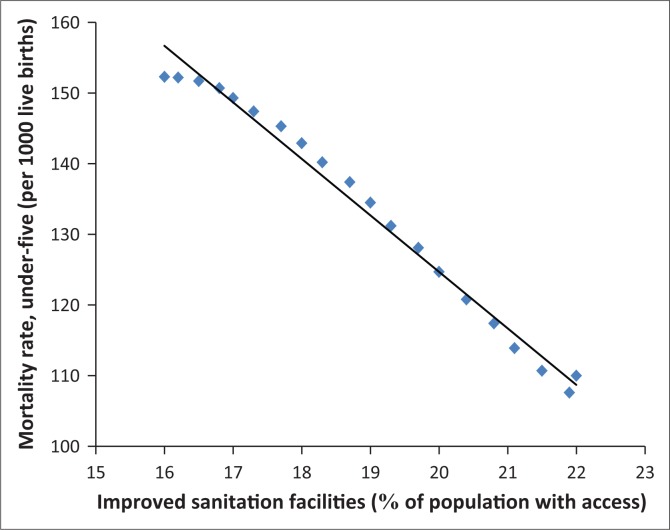
Improved sanitation and under-five infant mortality rate (per 1000 live births) in Cote d’Ivoire.

**FIGURE 7:**
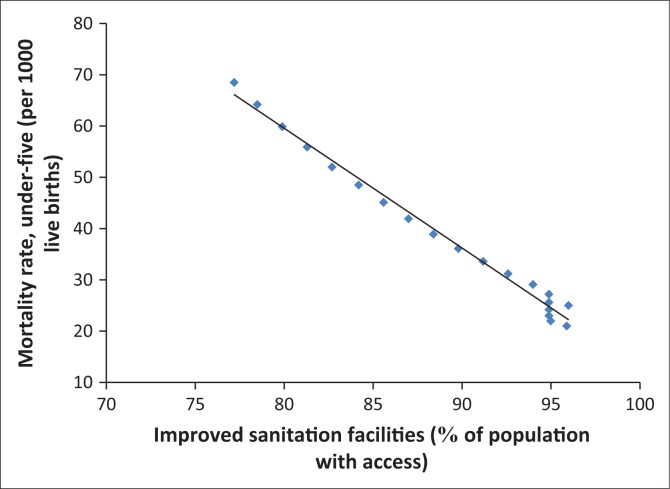
Improved sanitation and under-five infant mortality rate (per 1000 live births) in Egypt.

**FIGURE 8:**
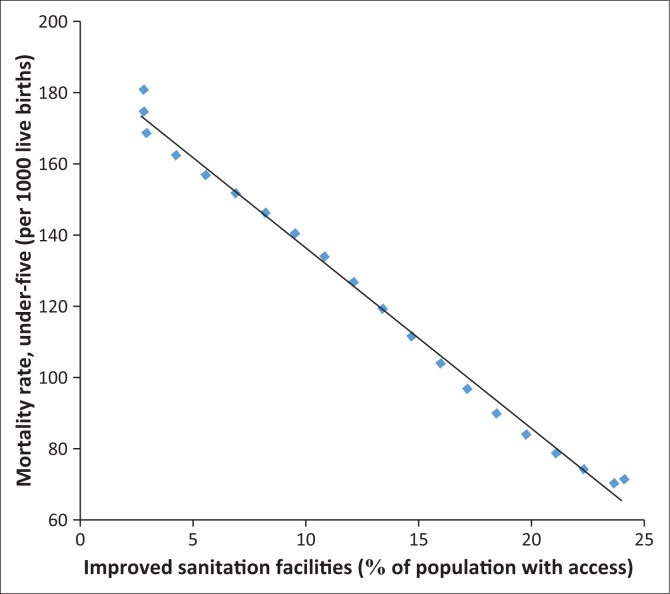
Improved sanitation and under-five infant mortality rate (per 1000 live births) in Ethiopia.

**FIGURE 9:**
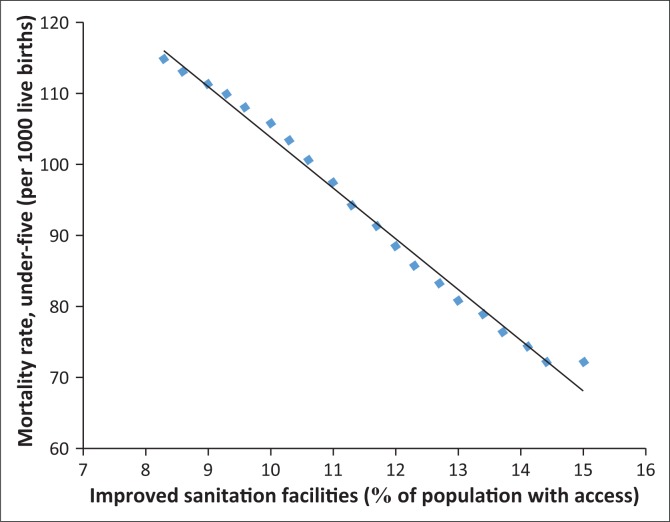
Improved sanitation and under-five infant mortality rate (per 1000 live births) in Ghana.

**FIGURE 10:**
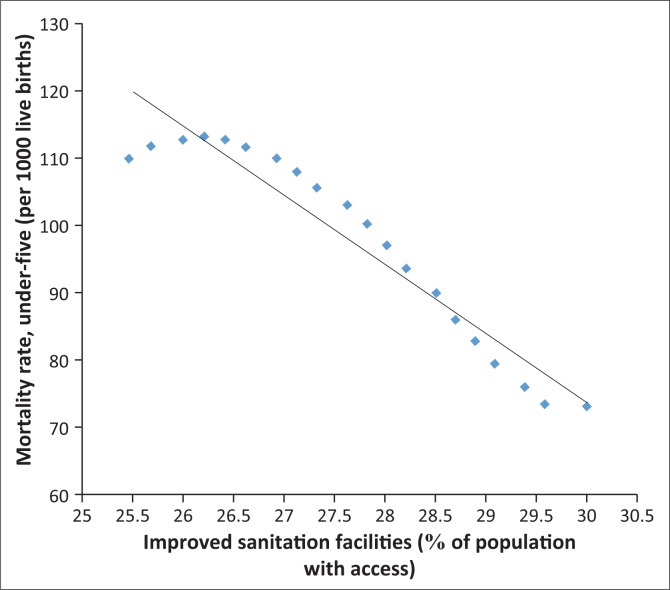
Improved sanitation and under-five infant mortality rate (per 1000 live births) in Kenya.

**FIGURE 11:**
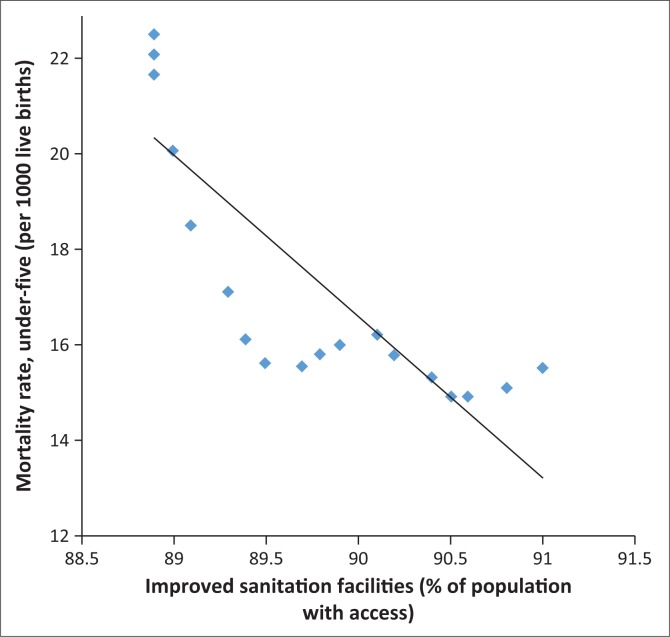
Improved sanitation and under-five infant mortality rate (per 1000 live births) in Mauritius.

**FIGURE 12:**
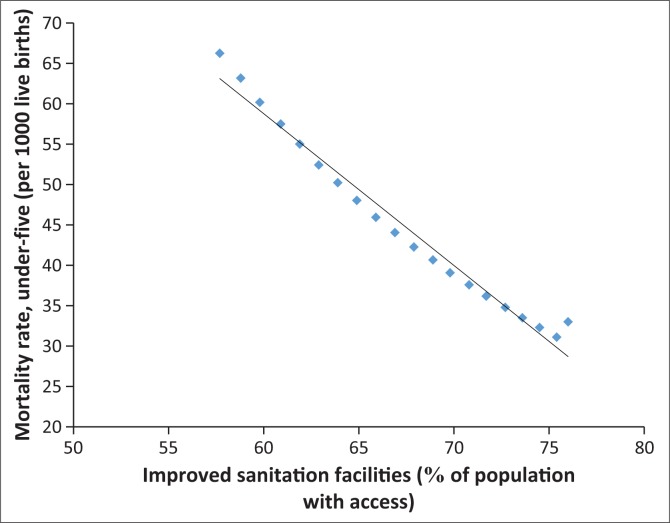
Improved sanitation and under-five infant mortality rate (per 1000 live births) in Morocco.

**FIGURE 13:**
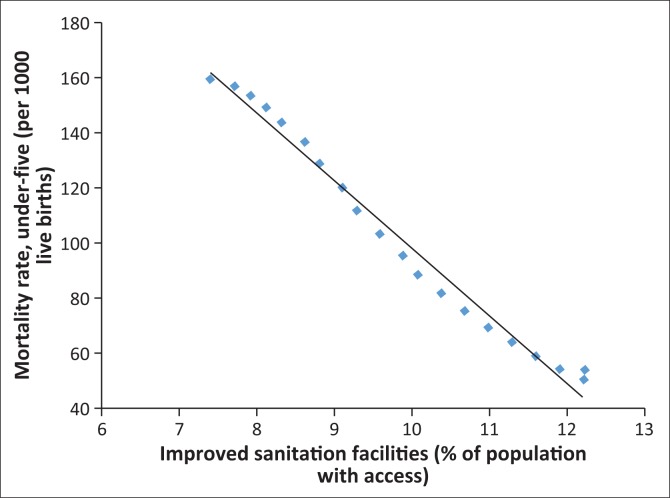
Improved sanitation and under-five infant mortality rate (per 1000 live births) in Tanzania.

## Empirical model and estimation techniques

The model to investigate the effects of improved sanitation on infant mortality in Africa was constructed for a panel of 33 African countries from 1994 to 2013 as follows:

INMit=β0+β1 ISANit+βj Σ Zit+αi+δt+εit [Eqn 1]

where index *i* refers to the unit of observation, *t* refers to the time period, *INM* refers to under-five infant mortality per 1000 live births, *ISAN* refers to improved sanitation expressed as percentage of population with access, *Z* refers to other control variables, *αi* refers to individual-specific unobserved factors, *δt* refers to time-specific unobserved factors and *εit* are individual- and time-specific residuals.

The Durbin–Wu–Hausman specification test that was developed in 1978 will help choose between fixed effect model (FEM) and random effect model (REM). The null hypothesis underlying the Hausman test is that individual effects are uncorrelated with the other regressors in the model.^16^ The test statistic has an asymptotic chi-square distribution and if the null hypothesis is rejected, a REM produces biased estimators, violating one of the Gauss–Markov assumptions; therefore, a FEM is preferred.^17^ Accordingly, the Durbin–Wu–Hausman specification test in this study has an asymptotic chi-square distribution of 463.1 that has strong evidence to reject the null hypothesis and confirms the suitability of FEM to be applied in this study (Table 2).

**TABLE 2:** Durbin–Wu–Hausman specification test results.

Specifications	Coefficients		(*b-B*) Difference	Sqrt (diag (V_b-V_B)) S.E.
	(*b*) Fixed	(*B*)		
Improved sanitation (percentage of population with access)	−1.9745600	−1.2042570	−0.7703986	0.0714940
Education expenditure or GDP	−3.1621890	−4.6405710	1.4783820	-
Women’s share of population (15+) living with HIV	1.2937000	0.9354393	0.3582609	-
Health expenditure or GDP	−2.5345570	−1.3486710	−1.1855860	0.2025830
GDP/C (PPP)	−0.0007516	−0.0009724	0.0002208	0.0000826
Population growth (annual)	0.2988438	0.1703583	0.1284855	0.3443518

*b*, consistent under Ho and Ha (obtained from xtreg); *B*, inconsistent under; Ha, efficient under; Ho (obtained from xtreg); sqrt, square root; diag, diagnostic; V, variance; S.E., standard error; GDP/C, gross domestic product per capita; PPP, purchasing power parity.

Test: Ho: difference in coefficients not systematic

Chi2(6)=(b−B) ′[(V_b−V_B)^(−1)](b−B)=463.11Prob>chi2=0.0000 [Eqn 2]

Accordingly, a panel data analysis using FE model has been conducted, and the results are shown in Table 3. In line with this, a White’s general test for heteroscedasticity was conducted and the result rejected the null hypothesis of homoscedasticity. Similarly, Wooldridge’s tests for autocorrelation in panel data were conducted and the null hypothesis that there is no first-order autocorrelation was not rejected. This implies that it was only heteroscedasticity but not serial correlation detected. According to Wooldridge,^18^ if heteroscedasticity is detected but serial correlation is not, then the usual heteroscedasticity robust standard errors and test statistics can be used. Accordingly, this study employed a FEM with corrected heteroscedasticity. In addition, to examine the consistency of results derived from FEM, another appropriate panel data estimation model called Prais–Winstein regression with corrected heteroscedasticity was conducted.

**TABLE 3:** Fixed effect regression with corrected heteroscedasticity.

Infant mortality under 5 years of age (per 1000 live births)	Coef.	S.E.	*t*	*P* > | *t* |
Improved sanitation (percentage of population with access)	−1.975	0.132	−14.93	0.000***
Education expenditure or GDP	−3.169	0.411	−7.69	0.000***
Women’s share of population (15+) living with HIV	1.294	0.163	7.91	0.000***
Health expenditure or GDP	−2.535	0.377	−6.72	0.000***
GDP/C (PPP)	−0.001	0.001	−1.77	0.078**
Population growth (annual)	0.299	0.440	0.68	0.497
Constant	240.416	10.825	22.21	0.000***
Number of observation	659			
Number of groups	33			
Observations per group	19			
*F* (6, 620)	155.93			
Prob. > Chi (2)	0.000			

Coef, coefficient; S.E., standard error; t, t-test; *P*, probability; GDP/C, gross domestic product per capita; PPP, purchasing power parity; *F*, F-test; prob., probability; chi, chi-square.

* refer the level of statistical significance at 10 percent level.

** refer the level of statistical significance at 5 percent level.

*** refer the level of statistical significance at 1 percent level.

## Regression results and main findings

Based on the Durbin–Wu–Hausman specification test and other specification tests discussed above, the first analysis task can be accomplished by using a ‘fixed effect model’. Subsequently, this study also employed another appropriate panel data estimation method known as ‘Praison–Winsten regression’ with corrected heteroscedasticity to verify the consistency of the results that will be revealed in using fixed effect estimation method.

Hence, the regression analysis using the FE model in Table 3 confirms that improved sanitation has been found to be the main influencing factor to reduce infant mortality rate in Africa with a statistical significance of 1% level. More precisely, the results from the FEM indicate that a 1% increase in improved sanitation accessibility for the population may reduce infants’ mortality under 5 years of age with a rate of about two infant deaths per 1000 live births. This implies that improved sanitation including access to clean water may dramatically improve the capacity to control infectious diseases such as typhoid and cholera transmitted by contaminated water, a major cause of illness and death in most African countries.

In line with this, the regression analysis from the FEM also indicates that if education expenditure as a ratio of GDP increases by 1%, infant mortality under 5 years of age might have been decreased by a rate of 3.2 infant deaths per 1000 live births. Likewise, if health expenditure as a ratio of GDP increases by 1%, infant mortality under 5 years of age might have been decreased by a rate of 2.5 infant deaths per 1000 live births. This implies that any effort for African countries to achieve a significant decline in infant mortality rate is highly linked to improvements in public sanitation and investments in education and health. Moreover, the regression result derived from the FE model confirm that economic growth measured in terms of growth in GDP/capita also matters for reducing infant mortality at 5% significant level.

In contrast, the regression results from Table 3 show that women’s share of population (15+) with HIV and population growth in African countries may strongly and directly affect infant mortality in African countries. For instance, if women’s share of population (15+) with HIV increases by 1%, this may cause of an increase in infant mortality under 5 years of age by a rate of 1.3 infant deaths per 1000 live births. That is because of the prevalence of mother-to-child transmittable diseases that are common in African countries where women often need to bear many children as a means of social recognition and economic survival. Some are also poorly educated and in many countries, age at marriage is still too low. As a result, women in Africa are highly exposed to HIV and other chronic diseases that may be transmitted from mother to child. Similarly, a 1% annual population growth may trigger an increase in infant mortality under 5 years of age by a rate of 0.3 infant deaths per 1000 live births. This is because of the fact that rapid population growth negatively impacts the economy such that governments may not be able to provide the required human capital investments for their population such as education, health and other physical infrastructures.

The results from the Prais–Winstein regression in Table 4 also confirm improved sanitation, investment in education and health and sustainable economic growth are statistically significant at 1% level to dramatically reduce infant mortality under 5 years of age. In line with this, the results from Table 4 also confirm that women’s share of population (15+) living with HIV and population growth have been found to be one of the important factors at macro level to exacerbate infant mortality under 5 years of age.

**TABLE 4:** Prais–Winsten regression with heteroscedastic panels corrected standard error.

Infant mortality under 5 years of age (per 1000 live births)	Coef.	Het-corrected std. err.	*z*	*P* > | *z* |
Improved sanitation (percentage of population with access)	−0.35428230	0.0418670	−8.46	0.000***
Education expenditure or GDP	−3.5678250	0.4036906	−8.84	0.000***
Women’s share of population (15+) living with HIV	0.4599310	0.0718049	6.41	0.000***
Health expenditure or GDP	1.1240610	0.0824517	13.63	0.000***
GDP/C (PPP)	−0.0016496	0.0001683	−9.80	0.000***
Population growth (annual)	−1.3785290	0.0591136	−23.32	0.000***
Constant	72.8020600	4.9202460	14.80	0.000
Number of observation	659			
Number of groups	33			
Observations per group	19			
Wald chi-2 (6)	1470.6			
Prob. > Chi 2	0.000			

Het-corrected std. err., heteroscedastic corrected standard error; *z, z*-test; *P*, probability; prob., probability; chi, chi-square.

* refer the level of statistical significance at 10 percent level.

** refer the level of statistical significance at 5 percent level.

*** refer the level of statistical significance at 1 percent level.

## Conclusion

The connection between access to improved sanitation and infant mortality has long been recognised. By the same token, a low infant mortality rate is a major contributor to increased life expectancy. Accordingly, infants’ major health threats and deaths are attributable to a number of infectious and parasitic diseases aggravated by lack of access to improved sanitation, and most could be avoided with the mass implementation of simple and low-cost interventions mainly through improving sanitation and hygiene. Of course, there has been a significant decline in infant death rates in many developing countries including some African countries. And yet, most of the sub-Saharan African countries are still having the highest rates of child mortality where one in eight children dies before age 5, which is more than 17 times the average for developed regions with a rate of one death in every 143 live births. Accordingly, this study reveals that improving sanitation would have a significant effect in reducing infant mortality in Africa in such a way that a 1% increase in improved sanitation accessibility for the population may reduce infant mortality with a rate of about two infant deaths per 1000 live births. In addition to improved sanitation, this study also confirms that a significant decline in infant mortality rate is highly linked to improvements in education and health, which implies that any effort in Africa to reduce infant mortality calls for huge investment in human capital, that is, education and health.

Moreover, the regression results derived from the FE model confirm that economic growth measured in terms of growth in GDP/capita also matters for reducing infant mortality at 5% significant level. In contrast, the study found women’s share of population (15+) with HIV and population growth in African countries may have strongly and directly affected infant mortality in most African countries where most women are poorly educated and are highly exposed to HIV and other chronic diseases that may be transmitted from mother to child. Last but not least, this study indicates that rapid population growth in Africa negatively impacts the economy which makes it more difficult for governments to provide the required human capital investments for their population such as education, health and other physical infrastructures, and ultimately to reduce infant mortality. Generally, the lesson from this study is that improved sanitation, investment in education and health, and better surveillance and monitoring of disease may significantly contribute to a remarkable decline in infant mortality in Africa as it has been already witnessed elsewhere, especially in east Asian countries. More specifically, improved sanitation is not only the most cost-effective public health interventions to save the lives of millions of infants but also can prevent economic losses associated with the direct costs of treating sanitation-related illnesses and ultimately increase income and economic growth through increasing productivity.

The findings have wide implications especially for African countries for which decreasing infant mortality is one of the most crucial priorities in the continent to reverse the current deep-rooted challenges related to human capital formation.
